# Rib plating of acute and sub-acute non-union rib fractures in an adult with cystic fibrosis: a case report

**DOI:** 10.1186/1756-0500-7-681

**Published:** 2014-10-01

**Authors:** Nathan C Dean, Don H Van Boerum, Theodore G Liou

**Affiliations:** Division of Pulmonary and Critical Care Medicine, Intermountain Medical Center, 5121 South Cottonwood Street, Murray, Utah 84107 USA; Division of Respiratory, Critical Care and Occupational Pulmonary Medicine, University of Utah, 26 North Mario Capecchi Drive, Salt Lake City, Utah USA; Departments of Surgery, Intermountain Medical Center, 5121 South Cottonwood Street, Murray, 84107 USA; University of Utah, Salt Lake City, USA

**Keywords:** Cystic fibrosis, Rib fracture, Rib plating, Bronchiectasis, Pain control

## Abstract

**Background:**

Rib fractures associated with osteoporosis have been reported to occur ten times more frequently in adults with cystic fibrosis. Fractures cause chest pain, and interfere with cough and sputum clearance leading to worsened lung function and acute exacerbations which are the two main contributors to early mortality in cystic fibrosis. Usual treatment involves analgesics and time for healing; however considerable pain and disability result due to constant re-injury from chronic repetitive cough. Recently, surgical plating of rib fractures has become commonplace in treating acute, traumatic chest injuries. We describe here successful surgical plating in a White cystic fibrosis patient with multiple, non-traumatic rib fractures.

**Case presentation:**

A-37-year old White male with cystic fibrosis was readmitted to Intermountain Medical Center for a pulmonary exacerbation. He had developed localized rib pain while coughing 2 months earlier, with worsening just prior to hospital admission in conjunction with a “pop” in the same location while bending over. A chest computerized tomography scan at admission demonstrated an acute 5th rib fracture and chronic non-united 6th and 7th right rib fractures. An epidural catheter was placed both for analgesia and to make secretion clearance possible in preparation for the surgery performed 2 days later. Under general anesthesia, he had open reduction and internal fixation of the right 5th, 6th and 7th rib fractures with a Synthes Matrix rib set. After several days of increased oxygen requirements, fever, fluid retention, and borderline vital signs, he stabilized. Numerical pain rating scores from his ribs were lower post-operatively and he was able to tolerate chest physical therapy and vigorous coughing.

**Conclusions:**

In our case report, rib plating with bone grafting improved rib pain and allowed healing of the fractures and recovery, although the immediate post-op period required close attention and care. We believe repair may be of benefit in selected cystic fibrosis patients, such as our patient who had suffered multiple rib fractures that were healing poorly.

## Background

Rib fractures associated with osteoporosis have been reported to occur ten times more frequently in adults with cystic fibrosis (CF) [1]. Fractures cause chest pain, and interfere with cough and sputum clearance leading to worsened lung function and acute exacerbations which are the two main contributors to early mortality in CF [2]. Usual treatment involves analgesics and time for healing; however considerable pain and disability result due to constant re-injury from chronic repetitive cough. Recently, surgical plating of rib fractures has become commonplace in treating acute, traumatic chest injuries [3]. Reported advantages include control of pain, and more rapid and complete healing of rib fractures [4]. In patients with CF, pain control may restore the ability to resume airway clearance, a critical element of maintenance therapy. In these patients, rib repair may be more effective than oral medications for controlling episodic but short-lived intense pain. We describe here successful surgical plating in a CF patient with multiple, non-traumatic rib fractures.

## Case presentation

**Figure 1:**
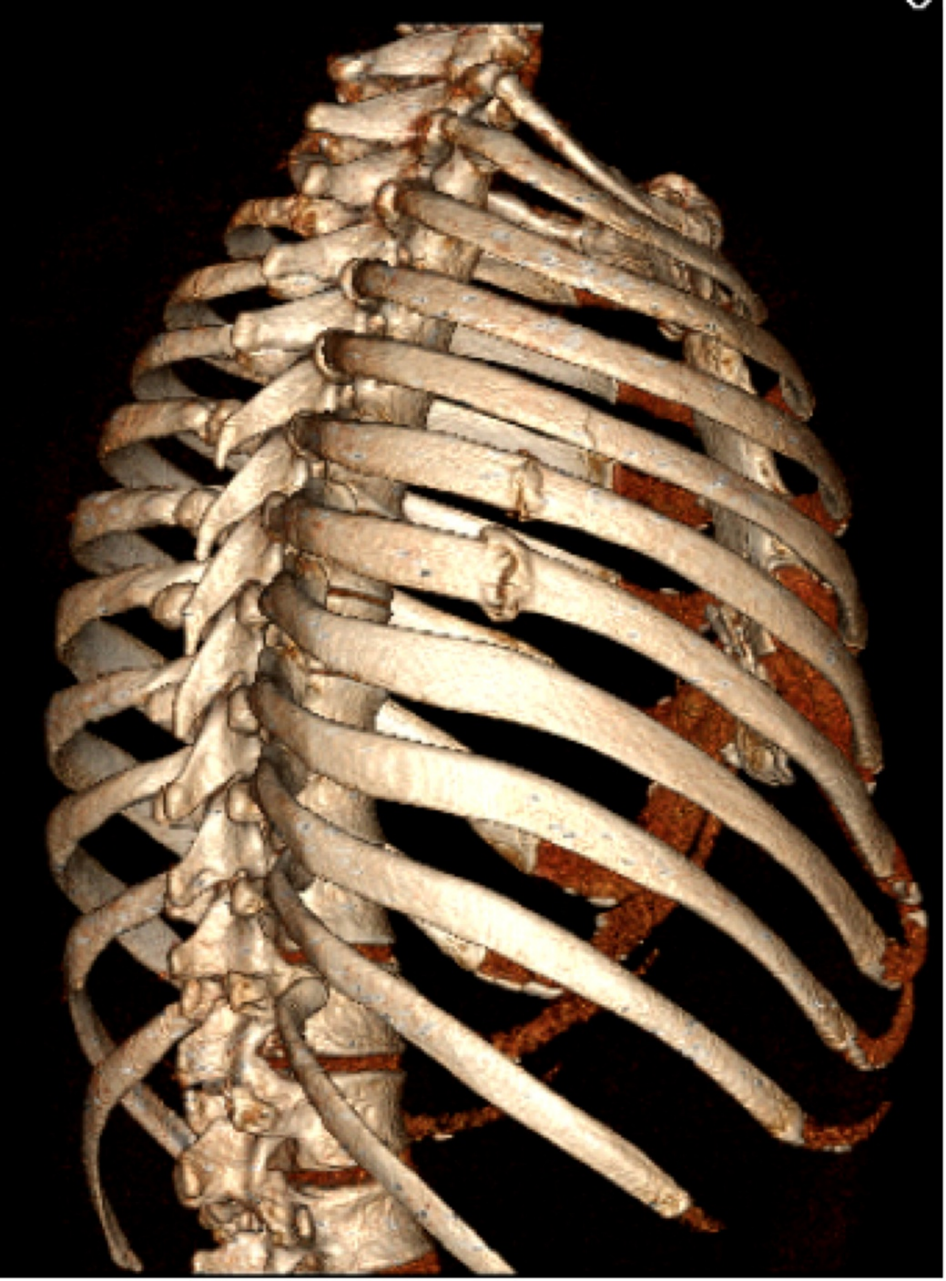
**Chest computerized tomography rib reconstruction shows 2 non-union subacute fractures, and the acute fracture in the adjacent 5th rib.**

A 37-year-old White male with CF identified at 3½ months, mutations F508del and R560T, was readmitted to Intermountain Medical Center for a pulmonary exacerbation. One week following discharge from his previous hospitalization 3 months earlier, he coughed hard and developed immediate focal pain in his right chest, mid to anterior axillary line at the 6th and 7th thoracic levels. He was seen in the emergency department and chest x-rays showed no abnormalities. He was treated symptomatically with oral analgesics; the pain waxed and waned but never resolved. A month later he had a particularly vigorous cough, and he felt a pop in the same area with even more pain. The pain worsened further just prior to readmission when his chest “popped” again while tying his shoe. A chest computerized tomography scan at admission (Figure 1) demonstrated an acute 5th rib fracture and chronic non-united 6th and 7th right rib fractures. He was taking chronic Vitamin D and calcium supplements and had a 25 hydroxyvitamin D2 and D3 level in the normal range (33 nanograms per milliliter). Forced expiratory volume in 1 second on admission was 0.66 liter (19% of predicted), and forced vital capacity 2.59 liters (61% of predicted), decreased from 0.80 (23% of predicted) and 2.73 (64% of predicted) at the end of his prior admission. His lungs are chronically infected with mucoid *Pseudomonas aeruginosa*. His body mass index at admission was 23; he had taken only a few short courses of corticosteroids over his lifetime.

Because of three contiguous fractures and the need for aggressive sputum clearance to treat his exacerbation, operative repair was recommended. An epidural catheter was placed both for analgesia and to make secretion clearance possible in preparation for the surgery performed 2 days later.

**Figure 2:**
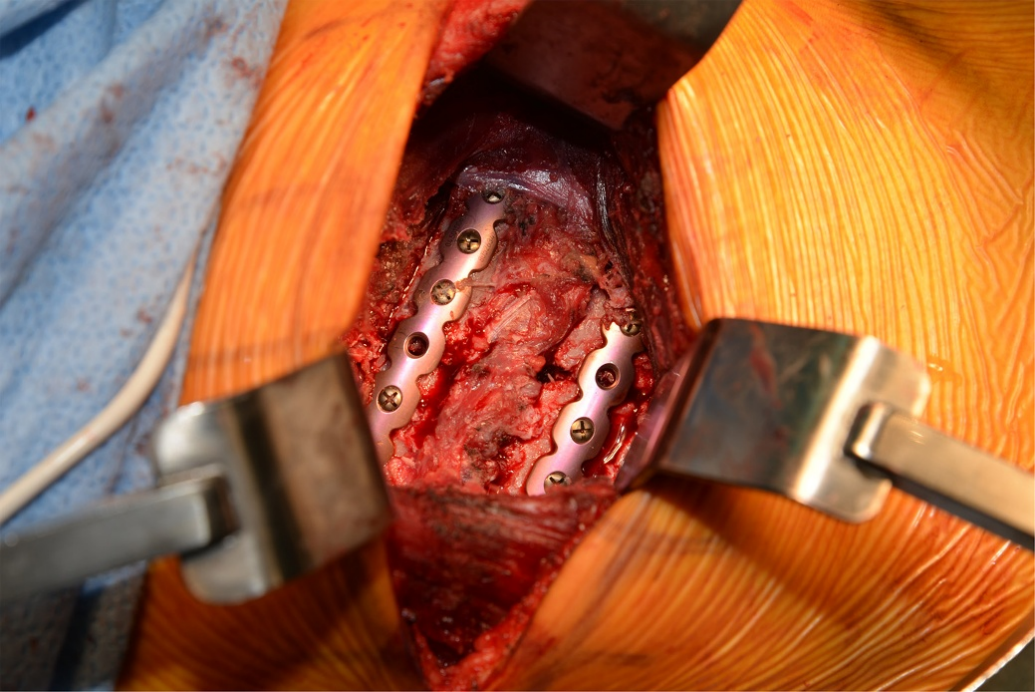
**Operating room photograph of plates bridging two of the rib fractures.**

Under general anesthesia, he had open reduction and internal fixation of the right 5th, 6th and 7th rib fractures with a Synthes Matrix rib set (Figure 2) [5]. The fracture sites were buttressed with iliac crest bone grafts. No chest tube placed, because the lung was attached to the parietal pleura likely from a prior, iatrogenic pneumothorax 13 years earlier that had required a chest tube. He was extubated in the recovery room and returned to a ward bed. After several days of increased oxygen requirements, fever, fluid retention, and borderline vital signs, he stabilized. Numerical pain rating scores are illustrated in Table 1 [6]. The fixated ribs were less painful to him post-operatively than the iliac crest bone harvest site. Bioavailable testosterone was low at 45.9 milligrams per deciliter (normal range 131–682), and replacement therapy was initiated. At discharge, lung function had plateaued with forced expiratory volume in 1 second 0.71 liter (21% of predicted) and forced vital capacity 2.26 liters (53% of predicted), but he was subjectively improved.

**Table 1:** **Numeric pain rating scale scores at 4 different time intervals during hospitalization, rated 0 to 10 with 10 being severe pain**

	Pain score ribs	Pain score iliac crest
Hospital admission, oral analgesics	5	Not applicable
Pre-operative, with epidural catheter	3-6	Not applicable
Post-operative day 3, with epidural catheter	1-2	4-5
Discharge home, oral analgesics	1-2	3-5

He gradually recovered from the surgery as an outpatient and resumed full time employment. He was readmitted 3 times over the subsequent year for acute pulmonary exacerbations, similar to his prior disease pattern. At the time of discharge one year post surgery, forced expiratory volume in 1 second was 0.89 liter (26% of predicted), and forced vital capacity 2.97 liters (79% of predicted), improved even compared with measurements over the year prior to the rib fractures. He has no pain or deformity in his right chest.

## Conclusions

Rib fractures are a common and significant cause of pain and morbidity in CF patients [1]. Rib fracture pain is difficult to treat with analgesics such as non-steroidal anti-inflammatory drugs and opiates because of the episodic and intense nature of cough. Rib pain impairs sputum clearance and leads to exacerbations of CF pulmonary disease, which may be the single most important factor leading to worsening lung function and prognosis. Poor healing and non-union of fractures may be more common in this patient group because of poor nutrition, low testosterone and vitamin D, and daily hard coughing.

In our case report, rib plating with bone grafting improved rib pain and allowed healing of the fractures and recovery, although the immediate post-op period required close attention and care. Surgical repair of fractures has been utilized primarily in trauma patients, particularly those with flail chests and respiratory failure. We believe repair may be of benefit in selected CF patients, such as our patient who had suffered multiple rib fractures that were healing poorly. Absent this surgery, we believe that his severe pulmonary exacerbation at the time of hospitalization would have been very difficult to treat successfully. Follow up at 1 year documented excellent functional outcome.

In the future, our patient might be considered for lung transplantation. Lung transplants are performed via median sternotomy, with access to the chest as well between the 5th and 6th interspace. Discussion with Utah lung transplant surgeons suggest that the presence of rib plates would not preclude the option of future lung transplantation, although fractures might occur at both ends of the titanium plates.

We recommend a randomized trial or at least case series of rib plating to treat multiple rib fractures in CF patients, and an attempt to determine whether bone grafting as an adjunct to the Matrix device is necessary.

## Consent

Written informed consent was obtained from the patient for publication of this Case Report and any accompanying images. A copy of the written consent is available for review by the Editor-in-Chief of this journal.

## Abbreviation

CF: Cystic fibrosis.
